# Effects of small interfering RNA targeting thymidylate synthase on survival of ACC3 cells from salivary adenoid cystic carcinoma

**DOI:** 10.1186/1471-2407-8-348

**Published:** 2008-11-26

**Authors:** Takashi Shirasaki, Shin-ichiro Maruya, Hiroki Mizukami, Seiji Kakehata, Hidekachi Kurotaki, Soroku Yagihashi, Hideichi Shinkawa

**Affiliations:** 1 Department of Otolaryngology, Hirosaki University School of Medicine, Hirosaki, Japan; 2 Department of Head and Neck Oncology and Surgery, International University of Health and Welfare Mita Hospital, Tokyo, Japan; 3 Department of Pathology, Hirosaki University School of Medicine, Hirosaki, Japan; 4 Department of Pathology, Odate Municipal Hospital, Odate, Japan

## Abstract

**Background:**

Thymidylate synthase (TS) is an important target for chemotherapeutic treatment of cancer and high expression of TS has been associated with poor prognosis or refractory disease in several cancers including colorectal and head and neck cancer. Although TS is known to regulate cell cycles and transcription factors, its potency as a therapeutic target has not been fully explored in adenoid cystic carcinoma (ACC).

**Methods:**

An ACC cell line (ACC3) was transfected with siRNA targeting the TS gene and inhibition of cell growth and induction of apoptosis-associated molecules were evaluated *in vitro*. In addition, the *in vivo *effect of TS siRNA on tumor progression was assessed using a xenograft model.

**Results:**

Our results demonstrated that ACC3 cells showed significantly higher TS expression than non-cancer cell lines and the induction of TS siRNA led to inhibition of cell proliferation. The effect was associated with an increase in p53, p21, and active caspase-3 and S-phase accumulation. We also found up-regulation of spermidine/spermine N1-acetyltransferase (SSAT), a polyamine metabolic enzyme. Furthermore, treatment with TS siRNA delivered by atelocollagen showed a significant cytostatic effect through the induction of apoptosis in a xenograft model.

**Conclusion:**

TS may be an important therapeutic target and siRNA targeting TS may be of potential therapeutic value in ACC.

## Background

Adenoid cystic carcinoma (ACC) is one of the most common salivary gland carcinomas and has indolent characteristics and an ultimately fatal outcome. Although ACC is thought to typically grow slowly, late occurrence of locoregional recurrence and distance metastasis is very common because of a propensity for perineural invasion. ACC shows various histological patterns including tubular, cribriform, and solid types. It has been widely accepted that the prognosis for tumors that predominantly comprise solid patterns is worse than that of those with a tubular or cribriform pattern [1]. Several molecules, including p16, p27, and E-cadherin, have been proposed as prognostic markers [2-4], but markers useful as therapeutic targets have not been identified in ACC.

Thymidylate synthase (TS), a folate-dependent enzyme, plays a crucial role in DNA biosynthesis. TS catalyzes the transfer of one methyl group from methylenetetrahydrofolate to generate deoxythymidine-5'-monophosphate (dTMP) from 2'-deoxyuridine-5'-monophosphate (dUMP). dTMP is then phosphorylated to 2'-deoxyuridine-5'-triphosphate (dTTP), which is a direct precursor for DNA. TS is a critical cellular target for 5-fluorouracil (5-FU), which is widely used in the treatment of several cancers including colorectal, breast, and head and neck cancer. The cytotoxicity of TS inhibitors, including 5-FU, has been attributed to the misincorporation of fluoronucleotides into DNA and RNA and the inhibition of DNA synthesis induced by the inhibition of TS enzyme activity. Consequently, the inhibition of TS activity has been shown to lead to apoptotic cell death resulting from intracellular thymidine depletion [5].

The role of TS as a target for its inhibitors and an oncogene is controversial and occasionally paradoxical. Overexpression of the TS gene has been associated with poor prognosis or disease refractory to 5-FU-based chemotherapy in head and neck squamous-cell carcinoma [6]. Although 5-FU exerts anti-proliferative effects by inhibiting TS expression, treatment with TS inhibitor has been shown to frequently induce up-regulation of TS expression in cell lines and tissues from patients treated with 5-FU [7,8]. In addition, TS overexpression or amplification induced by 5-FU exposure is associated with the acquisition of resistance to TS inhibitors [9-12]. Several recent studies have shown that TS gene induction leads to the acquisition of a malignant phenotype *in vitro*: transgenic mice overexpressing human TS in pancreatic islet cells developed islet hyperplasia or islet cell tumor [13,14]. On the other hand, we recently reported that simvastatin, one of the 3-hydroxy-3-methylglutaryl CoA (HMG-CoA) reductase inhibitors, exerted anticancer effects with down-regulation of TS expression [15]. These observations suggest that TS acts as a putative oncogene as well as a critical target for conventional and novel chemotherapeutic treatments.

There is some experimental evidence that the TS gene directly or indirectly affects cell proliferation and cell cycle regulators including the p53 pathway. E2F transcription factor, a cell cycle accelerator at G1/S phase, has been shown to induce expression of TS, proliferating cell nuclear antigen, and cyclin families in quiescent human cells and overexpression of TS results in down-regulation of tumor suppressor genes such as p53 and p21 in cancer cells [16-18]. Cancer cells transfected with mutant p53 have been shown to be more resistant to 5-FU treatment than parent cells with wild-type p53 [19]. Previous investigations have revealed that reductive expression of TS by antisense or small interfering RNA (siRNA) results in inhibition of cell proliferation or improvement in chemosensitivity [20-23]. However, the precise molecular and biological mechanisms of TS-specific inhibition or silence have not been fully elucidated. Although a recent investigation showed an intimate relationship between TS overexpression and poor clinical outcome in ACC, the significance of TS as a therapeutic target has been yet to be evaluated in ACC.

In recent years, RNA interference technology using siRNA has rapidly become a highly specific and powerful tool to silence target genes. To date, a number of studies have demonstrated that siRNA can be a novel tool for clarifying gene function in mammalian cells and may be applicable to gene-specific therapeutics. There have been no investigations into the efficacy of specific siRNA for the TS gene in the field of salivary gland cancer. Here, we have evaluated whether silence of the TS gene affects cell proliferation, cell cycle, and apoptosis in the ACC3 cell line.

## Materials and methods

### Cell culture

ACC3 cells were grown in DMEM supplemented with 10% fetal bovine serum, L-glutamine, penicillin, and streptomycin, and maintained at 37°C in a humidified atmosphere with 95% air and 5% CO_2_.

### Reagents and antibodies

Antibodies for immunoblotting were obtained from the following sources: mouse monoclonal anti-p21 (Santa Cruz Biotechnology, Santa Cruz, CA, USA); mouse monoclonal anti-p53 (Calbiochem, Darmstadt, Germany); rabbit polyclonal anti-active-caspase-3 (BioVision, Mountain View, CA, USA); mouse monoclonal anti-TS(Abcam, Cambridge, MA, USA); and mouse monoclonal anti-β-actin (Abcam).

### siRNA transfection

The targeted base sequence for human TS was 5'-GGATTCTTCGAAAAGTTGA-3' (Ambion, Austin, TX, USA). As a negative control, Silencer Negative Control #1 siRNA, a commercially available siRNA referred to as a nonspecific control (Ambion), was used. After incubation at 37°C for 24 h, cells were transfected with 50 nmol/L annealed siRNA oligos using siPORT™ lipid transfection agent (Ambion) following the manufacturer's protocol. Cells were treated with transfection agent/siRNA complex for 48 h and subjected to further analyses. For extraction of RNA and protein and flow cytometry analysis, cells were primarily plated at a density of 1 × 10^5 ^cells per well in 6-well plates.

### Cell viability assay

The effect of siRNA on cell numbers was determined by counting viable cells with a Cell Counting Kit-8 (Dojindo, Kumamoto, Japan). An equal number of cells (5000 cells/well) in 100 μL of culture medium were seeded into each well of a 96-well microplate and incubated for 24 h. Then, the cells were treated with TS siRNA or negative control siRNA. After incubation for 48 h, 10 μL of Cell Counting Kit-8 solution was added to each well and the plates were further incubated for 4 h at 37°C. Spectrophotometric absorbance was measured at 450 nm, with absorbance at 590 nm for reference.

### Cell cycle analysis

After treatment, cells were washed three times with phosphate-buffered saline (PBS), and collected by treatment with EDTA/trypsin solution. Collected cells were then fixed with 70% cold ethanol, incubated with RNase A (2 mg/mL in PBS), and stained with 50 μg/mL of propidium iodide (Sigma). Cell cycle data were acquired by FACScan equipped with Cell Quest software (Becton Dickinson, San Jose, CA, USA).

### Reverse transcription-PCR analysis

Total RNA from cultured cells was extracted using TRIzol (Invitrogen, Carlsbad, CA, USA) and 1 μg of total RNA was applied to one-step RT-PCR using a Gene Amp Gold RNA PCR reagent kit (Applied Biosystems, Foster City, CA, USA) according to the manufacturer's protocol. Oligomer primers were synthesized for TS (sense 5'-TCTGGAAGGGTGTTTTGGAG-3' and antisense 5'-CCTCCACTGGAAGCCATAAA-3') and glyceraldehyde-3-phosphate dehydrogenase (GAPDH) (sense 5'-CGAGATCCCTCCAAAATCAA-3' and antisense 5'-GTCTTCTGGGTGGCAGTGAT-3') (Sigma Genosys, Hokkaido, Japan). One-step cDNA synthesis and PCR amplification were performed at 25°C for 10 min; 42°C for 12 min; 95°C for 12 min; 35 cycles at 94°C for 20 s, each annealing temperature for 30 s, and 72°C for 30 s; and at 72°C for 7 min as a final extension. Annealing temperatures to amplify TS and GAPDH genes were 57°C and 60°C, respectively. PCR products were electrophoresed on 2% agarose gels and visualized by ultraviolet illumination.

### Real-time quantitative reverse-transcription PCR

For each sample, 1 μg of total RNA was retrotranscribed with the SuperScript first-strand synthesis system (Invitrogen, Carlsbad, CA, USA) in a 20 μL reaction. The obtained cDNA (10 μL) was then used for each duplicate in real-time quantitative reverse transcription-PCR, which was done on an ABI Prism 7700 Sequencing Detection System (Applied Biosystems, Foster City, CA, USA) using TaqMan Universal PCR Master Mix (Applied Biosystems) following the manufacturer's directions. RNA samples from human keratinocytes and human adult bronchial epithelial cells (Cell Applications, San Diego, CA, USA) were applied to real-time RT-PCR as control samples from non-cancer cells. PCR conditions were 50°C for 2 min and 95°C for 10 min followed by 40 cycles of 95°C for 15 s, and 60°C for 1 min. TaqMan probes for TS, GAPDH, and spermidine/spermine N1-acetyltransferase (SSAT) were from the Assays-on-Demand Gene Expression Assay Mix (Applied Biosystems). Assay ID numbers for TS, GAPDH, and SSAT were Hs00426591_m1, Hs99999905_m1 and Hs00161511_m1, respectively. The expressions of TS and SSAT genes were standardized using GAPDH as a reference gene, and relative expression levels for the panel of cell lines were quantified by calculating 2^-ΔΔC^_T_, where ΔC_T _is the difference in C_T _between target and reference genes.

### Western blot analysis

Cells were washed with PBS, and then incubated with medium containing agents or DMSO alone for control. After washing with PBS, the cells were then scraped with lysis buffer containing 20 mM Tris-HCl (pH 7.5), 150 mM NaCl, 1 mM Na_2_EDTA, 1 mM EGTA, 1% Triton X, 2.5 mM sodium pyrophosphate, 1 mM β-glycerophosphate, 1 mM Na_3_VO_4_, and 1 μg/mL leupeptin. After centrifugation, the supernatant was harvested as a total protein extract and stored at -80°C. Protein concentrations were measured using a protein assay reagent (Bio-Rad, Hercules, CA, USA). Equal amounts of protein (10 μg) were separated by gradient SDS-PAGE gels (Atto, Tokyo, Japan) and electrophoretically transferred to PDVF membrane (Atto). The membrane was blocked with 5% skim milk in PBS-0.1% Tween 20 at room temperature for 1 h. The membrane was incubated with primary antibody at room temperature for 1 h. Immunoreactivity was detected by sequential incubation with horseradish peroxidase-conjugated secondary antibody (Santa Cruz). Peroxidase activity was visualized by the enhanced chemiluminescence detection system (Amersham Biosciences, Buckinghamshire, UK).

### Xenograft models in nude mice

A total of 3 × 10^6 ^ACC3 cells were suspended in 100 μL PBS and injected subcutaneously into the right flank of 4-week-old female BALB/c *nu/nu *mice (Clea Japan, Tokyo, Japan). After 10 days, when the tumors had reached an average volume of approximately 100 mm^3^, mice were treated with TS siRNA or Silencer Negative Control #1 siRNA. For the *in vivo *transfection of siRNA, AteloGene™ (Koken Co., Tokyo, Japan) was used. siRNA-atelocollagen mixture was prepared according to the manufacturer's directions. 5 μM of siRNA mixed with 200 μM atelocollagen was injected into tumors. Tumor diameters were measured at regular intervals with a caliper, and the tumor volume in mm^3 ^was calculated by the formula: volume = length × width^2 ^× 0.5. All animals were treated in a humane manner and managed according to the guidelines of our animal facility. To detect apoptotic cells, a standard terminal deoxynucleotidyl transferase-mediated dUTP nick end labeling (TUNEL) assay was performed on formalin-fixed paraffin-embedded tissues according to the manufacturer's recommendations (S7101; Chemicon International, Inc., CA, USA). The number of positive cells in 1000 tumor cells within 4–6 microscopic fields at × 200 magnification was counted.

### Statistical analysis

Statistical analyses were performed by unpaired Student's *t *test using SigmaStat statistical software (version 3.1; Systat Software, Point Richmond, CA). All of the tests were two-sided. *P *< 0.05 was considered to be significant. Each experiment was repeated at least three times.

## Results

### Overexpression of TS mRNA in ACC3 cells

**Figure 1:**
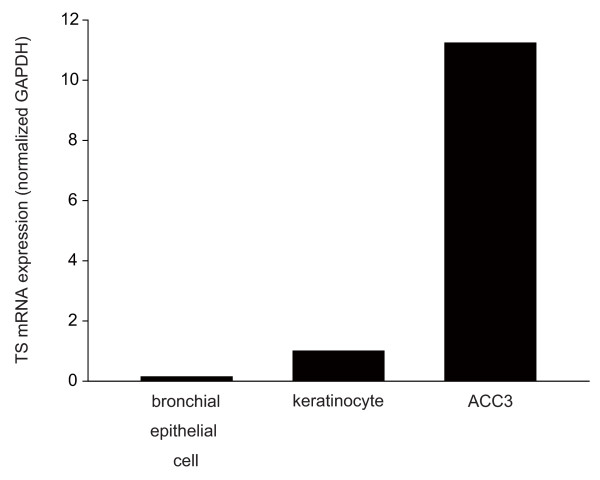
**Evaluation of TS mRNA transcript expression in the ACC3 cell line**. Expression was compared with that in keratinocytes and bronchial epithelial cells as normal cell controls using real-time RT-PCR.

Initially, the expression level of TS mRNA transcript was evaluated with real-time RT-PCR using RNA samples from control non-cancer cell lines derived from skin keratinocytes and bronchial epithelial cells. Expression in ACC3 cells was significantly higher than in the non-cancer cell lines. TS gene expression was 11-fold higher in ACC3 cells than keratinocytes (Fig. 1). When compared with human adult bronchial epithelial cells, the TS gene expression was increased 79-fold in ACC3 cells.

### Effect on cell survival of transfection with siRNA targeting TS

**Figure 2:**
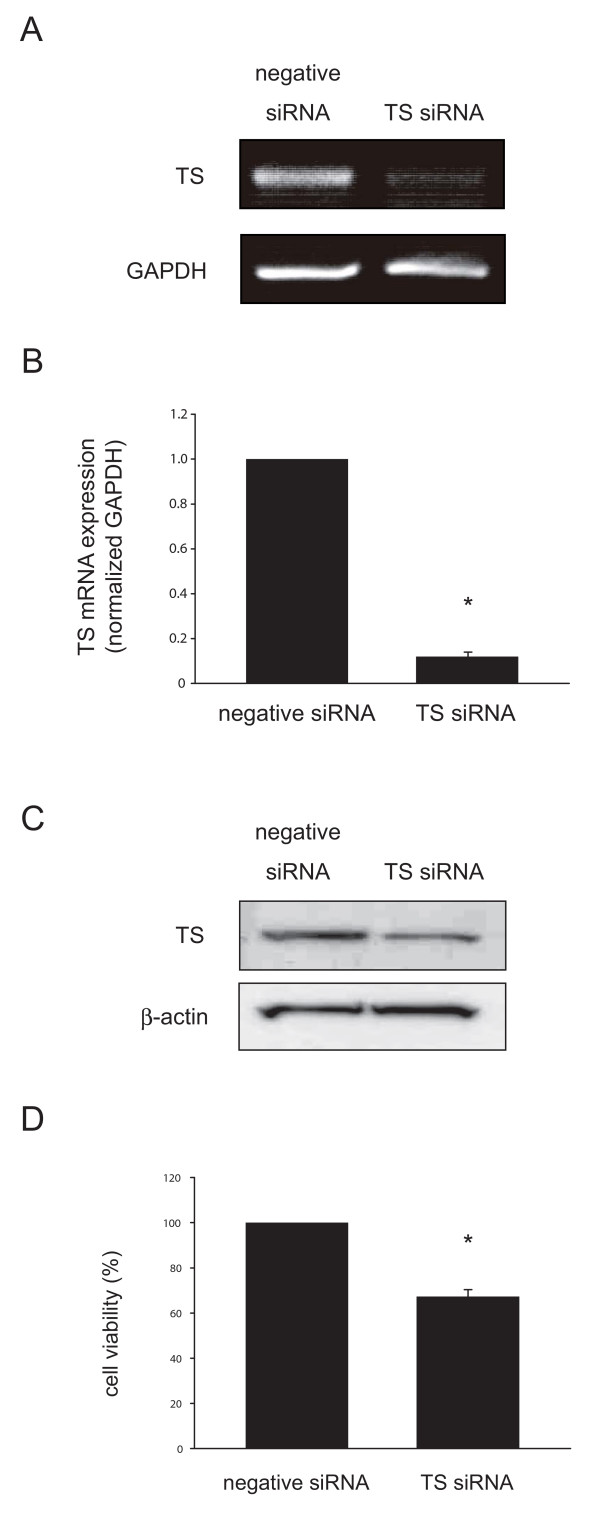
**TS expression suppressed by TS-specific siRNA in ACC3 cells**. After transfection for 48 h, TS expression was measured by conventional RT-PCR (A), real-time quantitative RT-PCR (B) and Western blot (C). Effect of TS suppression on cell survival in ACC3 cells (D). Cell survival conditions were evaluated by Cell Counting Kit-8 after transfection for 48 h. TS-suppressed cells were less proliferative than control cells (p < 0.01). The results are expressed as mean ± SD. *, *P *< 0.01, significantly different from control.

Next, we examined the effect of TS siRNA transfection on the viability of ACC3 cells. Transfection of cells with TS siRNA resulted in dramatically decreased TS expression (Fig. 2A). Real-time RT-PCR confirmed that the induction of TS siRNA decreased TS expression to 12% of the level in cells transfected with negative control siRNA (Fig. 2B). Western blot comfirmed effect of TS siRNA on protein expression (Fig. 2C) TS siRNA transfectant cells showed significantly reduced cell viability in comparison with control cells. Cell viability was inhibited by 67% in ACC3 (Fig. 2D).

### S-phase accumulation of cells by TS silence

**Figure 3:**
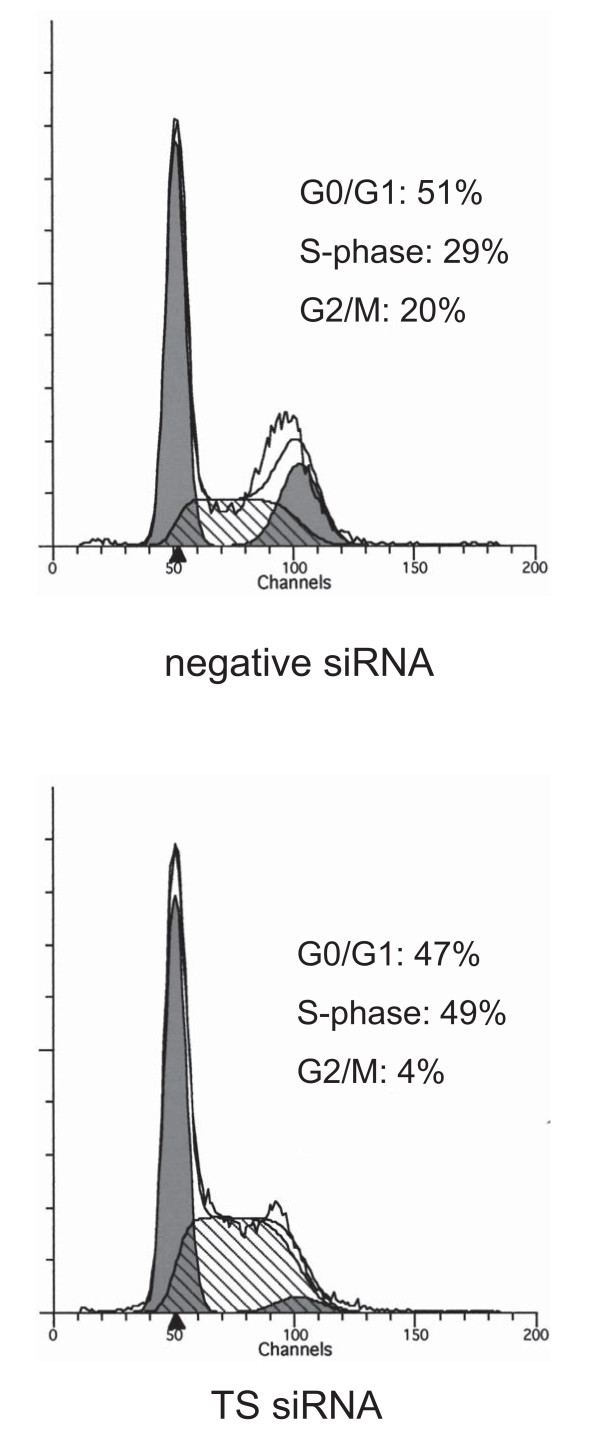
**Effect of TS suppression on cell cycle in ACC3 cells**. Representative flow cytometry graphs of cells 48 h after transfection with TS siRNA or negative control siRNA.

We examined whether TS inhibition caused by siRNA transfection affected the cell cycle. Cell cycle phases were determined by flow cytometry with propidium iodide staining. S-phase populations significantly increased, from 29% to 49%, in ACC3 cells after treatment with TS siRNA (Fig. 3). Experiments were repeated several times and identical results were obtained. These results indicate that TS siRNA leads to cell-cycle arrest at S-phase with a significant increase in the S-phase population.

### Effect of siRNA on the expressions of p53, p21, active caspase-3, and spermidine/spermine N1-acetyltransferase (SSAT)

**Figure 4:**
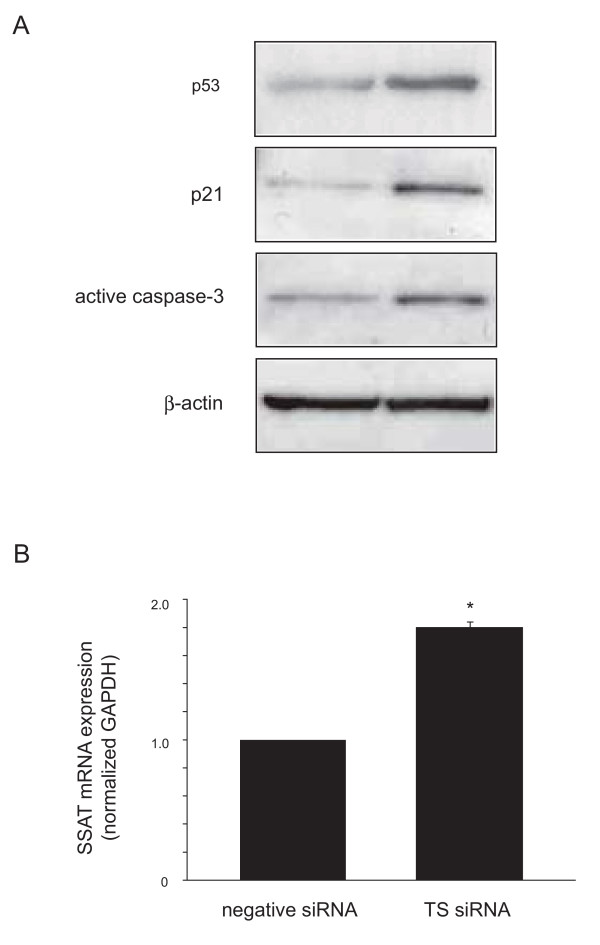
**Effect of TS silence on expression of p53, p21, active caspase-3, and SSAT**. Cells were transfected with each siRNA for 48 h and then protein and mRNA transcript expression was analyzed by Western blot (A) and real-time quantitative RT-PCR (B). The results are expressed as mean ± SD. *, *P *< 0.01, significantly different from control.

To assess the effect of TS suppression on the expressions of cell cycle- or apoptosis-associated molecules, we focused on the expression of molecules involved in p53 pathway and apoptosis. Western blot analysis revealed that cells transfected with TS siRNA showed up-regulation of p21 and active caspase-3 proteins (Fig. 4A). The antibody for p53 detects wild-type p53 protein. Real-time quantitative RT-PCR analysis demonstrated that the expression of SSAT mRNA transcript increased 1.8-fold in TS siRNA-transfected ACC3 cells (Fig. 4B).

### Inhibition of tumor growth by TS siRNA in ACC3 tumor xenografts

**Figure 5:**
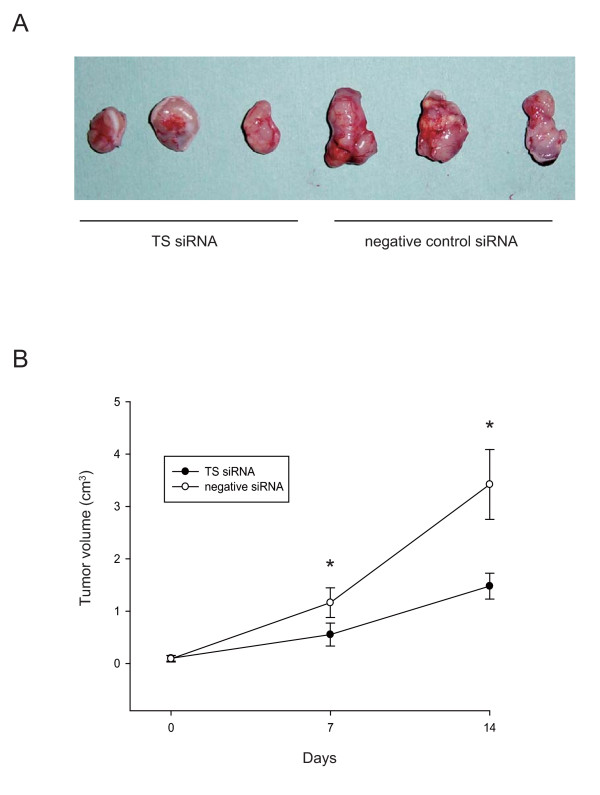
**An *in vivo *study of TS siRNA in ACC3 tumor xenografts**. 3 × 10^6 ^ACC3 cells were injected subcutaneously into the right flank of mice. When the tumors reached a volume of approximately 100 mm^3^, TS siRNA and negative control siRNA were injected to the tumors. TS and control siRNA were mixed with atelocollagen. Mice were sacrificed 2 weeks after siRNA injection. (A) Three representative xenograft tumors from each group are shown. (B) Tumor size was measured and tumor volumes calculated.  Each group consisted of six mice. The values are represented as mean ± SD. *, *P *< 0.01, significantly different from control.

**Figure 6:**
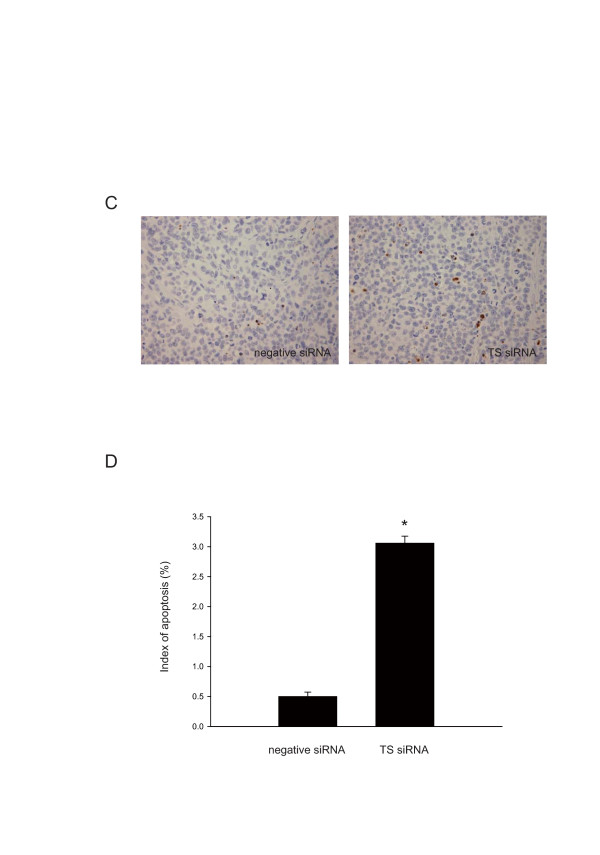
**An *in vivo *study of TS siRNA in ACC3 tumor xenografts**. 3 × 10^6 ^ACC3 cells were injected subcutaneously into the right flank of mice. When the tumors reached a volume of approximately 100 mm^3^, TS siRNA and negative control siRNA were injected to the tumors. TS and control siRNA were mixed with atelocollagen. Mice were sacrificed 2 weeks after siRNA injection. (C) After treatment with each siRNA, the xenografts were collected, fixed with 10% formalin, sectioned and stained for apoptotic cells by TUNEL method. The brown stain represented the DNA fragmentation of apoptotic cells and the blue stain showed the nuclei stain with hematoxylin (original magnification ×400). (D) The cumulative results showed the average number of apoptotic cells. Each group consisted of six mice. The values are represented as mean ± SD. *, *P *< 0.01, significantly different from control.

To investigate the antitumor effect of TS siRNA *in vivo*, we transfected TS siRNA and negative control siRNA into BALB/c *nu/nu *mice bearing ACC3 tumor xenografts. Subcutaneous tumors were developed by injecting 3 × 10^6 ^ACC3 cells into the right flank of female nude mice. When the tumor reached 100 mm^3 ^in volume, TS siRNA or negative control siRNA was injected and mice were observed for 2 weeks. The mean tumor volumes on day 14 were 1162 mm^3 ^in the TS siRNA-treated group and 3419 mm^3 ^in the negative control group. Significant differences between the two groups were found on days 7 and 14 (p < 0.01) (Fig. 5A and 5B). To assess the involvement of apoptosis in the suppression of tumor growth after TS silence, we conducted TUNEL assay and found significantly higher proportion of TUNEL-positive cells in TS siRNA-treated ACC3 cells (Fig. 6C and 6D).

## Discussion

Salivary gland carcinomas are uncommon neoplasms, accounting for approximately 5% of those arising in the head and neck region [24]. In general, these may be categorized into low grade and high grade malignancies based on their origin from either the terminal (intercalated) or the excretory ductal epithelium, respectively [25]. Biological and clinical classifications have been dependent on the histogenetic classification, but are occasionally diverse even within those categories [1]. Patients with higher histological grades appear to be more susceptible to locoregional recurrence and distant metastasis. However, effective treatment for unresectable tumors has not been established [26]. ACC is a representative salivary gland neoplasm originated from terminal (intercalated) epithelial cells and is characterized by indolent clinical course. However, most patients eventually succumbed to late-onset recurrence and distant metastasis. No effective therapeutic targets have been established because of its rarity.

We have shown that siRNA to down-regulate TS expression effectively inhibited cell proliferation and caused S-phase arrest in ACC3 cells derived from salivary gland carcinoma. We also confirmed that another TS siRNA targeting 5'-GGTGACTTTATACACACTT-3' decreased TS mRNA expression and cell viability to 23% and 78% of the level in ACC3 cells transfected with negative control siRNA, respectively (data not shown). Gene knock-down by siRNA is a highly effective approach to silence gene expression in experimental as well as therapeutic setting. However, the interpretation of data is occasionally complicated because of non-specific gene silence, so-called off-target effects, which results from unintended interactions between silence molecule and cellular components and inappropriate concentration of siRNA [27]. Our TS-siRNA effectively suppressed both mRNA and protein. In cDNA microarray analysis in a squamous cell carcinoma cell line, our TS-siRNA primarily silenced TS mRNA expression (manuscript in preparation).

Previous reports have shown that 5-FU treatment leads to S-phase accumulation in colorectal cancer cells *in vitro *and *in vivo *[28]. In addition, antisense oligodeoxynucleotides to down-regulate TS have been shown to induce S-phase arrest in a murine colon carcinoma cell line and human cancer cell lines [22,23]. Our data demonstrate that TS silence predominantly targets the S-phase transition; thus, TS has a critical role in the regulation of the S-phase checkpoint. S-phase arrest by TS inhibition may be regulated by various mechanisms. The present cell cycle analysis showed that transient silence of TS gene induced S-phase accumulation without increase in sub-G1 population in ACC3 cells. The mechanism of S-phase accumulation induced by TS inhibition has not been fully understood. However, S-phase arrest seemed to be a common phenomenon of TS inhibition. TS inhibitors, such as 5-FU and ZD9331, induced increase in sub-G1 population or apoptosis after S-phase accumulation [8,29]. Since the present TS siRNA induction into ACC3 cells is temporal or transient, S-phase accumulation with activation of p53 pathway may be considered to be an early event in pro-apoptotic effect of TS inhibition.

We presently focused on the interaction between TS silence and apoptotic mechanism. Previous studies have shown that overexpression of TS gene leads to down-regulation of p53 or p21 and the treatment with TS inhibitors leads to induction of p21 [29]. We have demonstrated here that targeted suppression of TS expression increased expression of p21 and active caspase-3 along with up-regulation of wild-type p53 in ACC3 cells. Our present results appeared to be consistent with previous studies showing that TS inhibition is associated with S-phase accumulation and p21 activation [22,23,28]. Our data that TS silence led to p21 up-regulation seemed to be controversial, because p21 activation correlated with G1 arrest in cell cycle. This discrepancy implies that different cell cycle mediators play a certain role to control the interaction between p21 and S-phase arrest induced in TS inhibition.

Here, real-time RT-PCR analysis demonstrated TS mRNA transcript to be significantly up-regulated in ACC3 cells compared with non-cancer cell lines. Numerous investigations have focused on the relationship between TS expression and resistant activity to TS inhibitory agents. Comparative genomic hybridization analyses have revealed that chemoresistance to 5-FU or poor clinical outcome is associated with gain of the 18p11.32 region encoding the TS gene [9]. Immunohistochemical study also showed that overexpression of TS protein was commonly observed and associated with resistance to chemotherapy in head and neck squamous cell carcinoma [6]. In the meantime, we found significant amplification of TS mRNA transcript in ACC3 cells, which were derived from adenoid cystic carcinoma of the parotid gland. This cell line has been widely used in a number of studies as a characteristic research model of ACC [30]. Chikamatsu *et al*. [31] recently reported that high expression of TS protein was observed in approximately one third of ACC cases and was possibly associated with poor clinical outcome. We have demonstrated here that TS siRNA effectively inhibited tumor growth through the activation of apoptosis in an *in vivo *xenograft model. Although the efficacy of conventional chemotherapeutic agents on salivary gland cancer has been regarded to be limited, TS may be an important target for the treatment of ACC.

We demonstrated that TS silence by specific siRNA induced the overexpression of spermidine/spermine N1-acetyltransferase (SSAT) in ACC3 cells. SSAT is a gene involved in polyamine metabolism. Polyamines (putrescine, spermidine, and spermine), derived from ornithine in the urea cycle, play important roles in cell growth and cell differentiation by modulating DNA structure, DNA replication, and DNA repair. SSAT is a major enzyme in the polyamine degradation pathway [32]. We focused on the relationship between TS silence and SSAT expression because there have been several reports that polyamine metabolism is involved in cellular reaction induced by chemotherapeutic cytotoxic agents, such as TS inhibitors. It has been reported that treatment with chemotherapeutic agents such as 5-FU and cisplatin induces up-regulation of SSAT expression in several cancer cell lines and N^1^, N^11^-diethylnorspermine, a polyamine analog, enhances cytotoxic effect of 5-FU and oxaliplatin along with up-regulated SSAT expression [33-35]. In addition, overexpression of SSAT has been shown to induce cell death or apoptosis. Increased expression of SSAT yields depletion of cellular polyamine content and consequently inhibits cell survival [36]. The detailed mechanisms and involvements of these molecules in TS silence mechanism are under further investigation.

In summary, our study showed that siRNA targeting TS efficiently reduced TS expression with inhibited cell growth in ACC3 cells from salivary adenoid cystic carcinoma. The cytostatic activity of TS silence appeared to be associated with cell cycle arrest at S-phase and activation of p53 pathway. Furthermore, we showed that TS siRNA delivered by atelocollagen exerted an anti-proliferative and apoptotic effect in a xenograft model. Further elucidation of the role of TS as an oncogene or therapeutic target may contribute to the development of optimal treatments for salivary gland carcinomas including ACC.

## Competing interests

The authors declare that they have no competing interests.

## Authors' contributions

SM and TS coordinated the study, performed *in vitro *and *in vivo *experiments and statistical analyses, and drafted the manuscript. HK and HM performed histological evaluation and TUNEL assay in *in vivo *analyses. SK contributed to conception and design of study and interpretation of data. HS and SY participated in design and coordination of study and helped to draft the manuscript. All authors have read and approved the final manuscript.

## Pre-publication history

The pre-publication history for this paper can be accessed here:


